# The Education Pipeline of Biomimetics and Its Challenges

**DOI:** 10.3390/biomimetics7030093

**Published:** 2022-07-07

**Authors:** Shoshanah Jacobs, Marjan Eggermont, Michael Helms, Kristina Wanieck

**Affiliations:** 1 Department of Integrative Biology, Department of Management, University of Guelph, Guelph, ON N1G 2W1, Canada; sjacob04@uoguelph.ca; 2 Department of Mechanical and Manufacturing Engineering, Schulich School of Engineering, University of Calgary, Calgary, AB T2N 1N4, Canada; meggermo@ucalgary.ca; 3 School of Mechanical Engineering, Georgia Institute of Technology, Atlanta, GA 30318, USA; michael.helms@me.gatech.edu; 4 Faculty of Applied Informatics, Deggendorf Institute of Technology (DIT), Teaching Area Biomimetics and Innovation, Grafenauer Str. 22, 94078 Freyung, Germany

**Keywords:** teaching, education, STEM, training K-12, higher education, industry training

## Abstract

Biomimetics must be taught to the next generation of designers in the interest of delivering solutions for current problems. Teaching biomimetics involves teachers and students from and in various disciplines at different stages of the educational system. There is no common understanding of how and what to teach in the different phases of the educational pipeline. This manuscript describes different perspectives, expectations, needs, and challenges of users from various backgrounds. It focuses on how biomimetics is taught at the various stages of education and career: from K-12 to higher education to continuing education. By constructing the biomimetics education pipeline, we find that some industry challenges are addressed and provide opportunities to transfer the lessons to application. We also identify existing gaps in the biomimetics education pipeline that could further advance industry application if a curriculum is developed.

## 1. Introduction

Biomimetics, as the “interdisciplinary cooperation of biology and technology and other fields of innovation”, aims to solve practical problems [1] using our knowledge of biological systems. Because of the appeal of looking to natural systems for potential solutions, there is a breadth of people all different in motivation, experience, and training, who are engaging in biomimetic practice [2]. On the one hand, these include biomimeticians [3] educated in biomimetics specifically. On the other hand, and in the majority, this includes biologists, engineers, architects, and designers, in academic, industrial, and governmental settings, using biomimetics as a design methodology with no or limited formal education specific to biomimetic practice. These informal practitioners have likely interacted with the topic during their educational career, either as a “sneak-peak’’ in K-12 or higher education or during external additional qualifications (e.g., [2]), and most of the time, they work together in inter- and transdisciplinary projects or teams [4]. In this paper and in the context of biomimetics, interdisciplinarity describes the cooperation of different unrelated academic disciplines to cross boundaries and synthesize links between disciplines to create new biomimetic knowledge [5,6]. Transdisciplinarity means the cooperation between different sciences and the engagement of non-academic partners in the process [5,6], like practitioners from industry transcend traditional boundaries and create new biomimetic knowledge and solutions.

Because biomimetics is a superdiscipline, one that integrates multiple traditional disciplines, to advance the practice, formal pedagogies for teaching the unique knowledge, methods, and values of biomimetics must be developed, tested, and taught to practitioners and would-be practitioners of biomimetics [7]. However, there are still various aspects of biomimetics instruction and pedagogy under investigation: what and whom to teach ([8]), whom to involve in projects [9,10,11], which resources, tools, and methods to include in teaching and implementing biomimetics [7,12,13], and when and how to offer learning opportunities [2,8]. Additionally, the key concepts of educational programs need to be clarified, e.g., the effectiveness of knowledge translation and transfer of biology to design. Only if what is needed to go from knowledge to action is clearly understood can biomimetics take deeper root in design and problem solving to address our 21st-century challenges. We must also continue to identify pathways by which successful, sustainable technologies are developed, and determine the means by which the understanding of biomimetics and biological systems can be taught to researchers, practitioners, and students who are not trained in all of the relevant disciplines. Finally, we must teach biologists how to study and characterize biology in ways that provide utility to design teams, how to communicate with designers, and how to make use of their fundamental knowledge of systems of biology for the development of sustainable systems-oriented solutions to human problems; a role which offers new job perspectives for biologists as a critical member of a design team (e.g., [14]). Engineers, experts, and designers with this biomimetic knowledge will become part of the future skills labor force ([15]). This is why education on this topic is needed.

### 1.1. Motivation

Earth is reaching and, in most categories, exceeding its planetary boundaries. In the G20 countries, the sustainable limit for all but one boundary (water) has been exceeded (https://goodlife.leeds.ac.uk/countries/#G20; accessed on 25 April 2022). Ellis and Ramankutty (2008) predicted that we would soon be living in a world where less than 25% of our planet’s ice-free land is unaffected by human activity, and in 2020, the United Nations gave us 12 years to act [16]. We are now beginning to experience how crossing our planetary boundaries concerning climate change, biochemical flows, land-system change, and biosphere integrity strains the resilience of our systems [17]. If we continue on our current course, humans and the biosphere in which we live will change permanently [18,19,20,21,22] and, critically, at a rate unlikely to support healthy adaptation.

There are at least two productive human responses concerning global ecological change. The first is to prevent or reduce the harmful effects that human activity is having on the planet’s climate. By reducing the impact of industrial activity on the environment, we can allow the planet to recover. Months of worldwide lockdowns during the COVID-19 pandemic have shown that certain forms of recovery are possible at paces much faster than studies have suggested (marine systems: [23]; biodiversity: [24]; water quality: [25]; review: [26]; but see [27]). To reduce the harmful effects of human activity, we need to find new sustainable ways of manufacturing in all phases of product development. Though there are several ways in which we might achieve this, one promising approach is to model how biological systems overcome challenges in production and emulate the design principles under which they evolved [28]. Recent federal government investment in circular economy research and implementation is an example of this response type, with its emphasis on ‘nature-based solutions’ [29]. The second way we might respond to the climate crisis is to develop systems to replace the ecological services on which we rely. For example, while much work has been done to reverse the loss of pollinator species by developing conservation strategies [30], researchers are also studying how to replace biological pollinators with technological ones. Both types of responses require designers to know about biological systems. This can be achieved by supporting designers, also in industry, to integrate innovative ideas and technologies with relevant biomimetic methodologies and concepts [31]. Therefore, developing new methods of production or new technologies that support life on Earth (e.g., [32,33]) shifts the superdiscipline of biomimetics from a desirable means of technological development to an obligate means by which society, including industry, will adapt to the climate crisis.

Biomimetics, (a.k.a. Biom*—a term used to represent and acknowledge the many facets and philosophies within the superdiscipline of biomimetics [7]), was predicted to become a dominant paradigm for engineering and other technological disciplines [34,35] with great potential for scientific [14,36], societal [37,38], and economic impact [39,40]. Using biom* to meet pressing and global environmental challenges will require that more people from all disciplines and across all industrial sectors (1) know how to work well together on transdisciplinary teams, and (2) understand biological systems, their development, and evolutionary adaptation [9], as well as how they relate to the human environment.

### 1.2. Biomimetics and Education

To successfully focus our attention on the teaching of biology and of knowledge translation and transfer, and to not get “lost in knowledge translation” [41], we must study how non-biologists learn about nature and use biological information, how it is understood and/or misunderstood, and how it is used, shared, and valued so that we can integrate the use of biological systems knowledge into design, and support a culture change in the next generation of designers to include nature-inspired solutions. With a growing number and increasing experience of professional biomimeticians [3], those trained in biomimetics specifically and with knowledge of biology and design, there is an opportunity to describe the biomimetics education pipeline fully. If successful, we will be addressing one of the big challenges in Biom*, that is, “to educate new generations of would-be-designers in the paradigm of biologically inspired design” who address real problems [42] (p. xiii). If we consider industry to be a crucial part of addressing climate change, the topics taught in the pipeline need to address industry needs. So that a new generation of designers will include current employees in industry who attend post-secondary training as well as students who go through the whole pipeline and have the chance to learn the topic at various stages with deep knowledge of the biomimetic process and the underlying biology.

**Figure 1:**
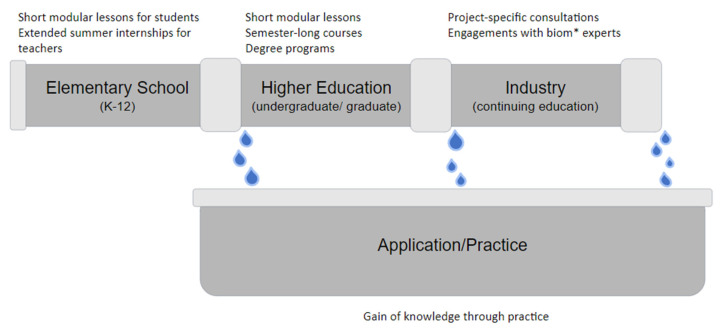
The biom* education pipeline.

Figure 1 shows that currently, the teaching of biomimetics and/or biomimicry within systems of education is informal at the K-12 level and sparsely distributed in post-secondary education. Globally, there are no government-mandated curricula in biomimetics in grade school curricula, except for an instance in Germany, where biomimetics is mentioned in curricula for several student groups (https://www.gym8-lehrplan.bayern.de/contentserv/3.1.neu/g8.de/id_26433.html; accessed on 20 November 2021 [8]). Usually, teachers who introduce it to their students do so to ‘enrich’ learning rather than incorporate it into the core curriculum. Within post-secondary education, several institutions offer complete graduate degrees in biomimetics, and this number is growing. These programs often bridge disciplines through their foundations, usually within engineering departments, that focus the research agenda in biomimetics on knowledge translation rather than mobilization.

At the heart of our challenge, we find an age-old problem: our education systems are not designed to meaningfully value transdisciplinary teaching and learning within the core curriculum. Though many institutions and boards of education worldwide have stated that transdisciplinary teaching and learning are valuable, even critical to a student’s education, very few have embraced these skills within the core offering [43]. Extra-curricular, co-curricular, and work-integrated programs largely exist where teaching and learning about biom* occurs within undergraduate education, and only a handful of graduate degree programs exist with an emphasis on biom* training [2] (but see [44,45] for examples of elective courses).

Yet, the development of education programs across sectors, from formal graduate education to workshops offered by for-profit organizations, if considered together, may offer solutions to some of the long-standing challenges faced by proponents and practitioners of biom* (e.g., [46,47,48]). In this paper, we begin by summarizing the state of biom* education in each of these sectors, i.e., K-12 education, undergraduate, graduate higher education, as well as extra-formal education, like training. Then we assemble a list of challenges from industry using a variety of sources. We then deduce their educational implications and seek examples of sectors or practices that address these challenges in hopes of connecting problems to potential solutions.

## 2. The Biom* Education Pipeline

Biomimetics can significantly contribute to education and training for various target groups along the educational pipeline [8]. While biomimetics is taught in various depths from kindergarten to university and as an additional qualification for practitioners in industry, there is no common understanding of how and what to teach in the different phases to educate this needed next generation. To keep students interested in the topic and to motivate them to gain knowledge and expertise in this field, biom* education can learn from STEM (science; technology; engineering; mathematics) experiences more broadly, as there is too little data on the specific biom* educational pipeline yet. Maltese et al. (2011) recommend that science education needs to be personal, more related to the lives of students, i.e., relevant and local, engaging students with real-world problems [49]. Biomimetics naturally connects to current societal challenges, such as sustainability, climate change, pollution, and biodiversity, as well as to improving products that students use in their daily lives. Introducing biomimetics to students will increase personal, social, and professional skills, including interdisciplinary thinking, personal commitment and communication skills [8]. Additionally, discussing job opportunities in biom* will foster student engagement and raise their career awareness, which is a major challenge in biomimetics. The qualification of students holding a degree in biomimetics is hard to define by industry representatives, which makes finding a job more difficult [50]. It is imperative that the development of the biomimetic educational pipeline and associated biomimetics curricula, learning experiences, and programs empower students to address the needs and expectations of industry; and further that these programs communicate to industry the benefits of such training.

### 2.1. Biom* Training in K-12

At the intersection of biology and engineering, many of the goals associated with introducing K-12 students to biom* topics are similar to those of encouraging students to explore pathways in STEM more broadly. As such, federally supported funding opportunities exist to offer curricular enrichment for elementary and high school students. A review of 8 such programs (Table 1), mostly based in the United States and funded through the National Science Foundation, demonstrates the types of K-12 biom* educational models currently available.

The K-12 biom* education programs are available in three general formats: (1) direct interventions with K-12 students, (2) K-12 teacher training, and (3) programs that do both. The programs designed for direct contact with students span between a single day to 6 weeks, with hands-on activities for students who travel to a central location and work together in small groups, often on a design challenge guided by a facilitator who is usually not their own teacher. These programs often focus on students from lower socioeconomic communities and are designed as curriculum enrichment activities. The teacher training programs are offered during the summer professional development period as continuing education. They are designed to inspire teachers to incorporate biom* and STEM elements into their existing curriculum and to provide the content and process knowledge necessary to either develop their own biom* lessons or to deploy specific existing lessons. The BIRDEE program provides both a high school engineering classroom curriculum and summer teacher training.

The reported outcomes of these programs are varied. Both student and teacher groups enjoy these interventions and opportunities for enriched education. With respect to evidence of learning, students report learning more about design thinking and collaboration rather than the skills associated specifically with biom* (e.g., [55]). Similarly, teachers report learning about the value of maker spaces for hands-on learning or design thinking approaches (e.g., [57]). Though participants reported a greater understanding or awareness of biom*, because the programs only lasted from 1 day to 6 weeks, biom* was more of a context for experiential learning rather than the immediate focus. These short-duration programs, though clearly beneficial for increasing awareness and engagement in STEM topics generally, do not afford the time and immersion required to fully experience a biom* design process. Careful attention to learning goals and available resources must be paid (e.g., [51]).

#### 2.1.1. BIRDEE, Georgia Institute of Technology

In 2020, Helms, with the Center for Biologically Inspired Design and Center for Education Integrating Science Mathematics and Computation, both at Georgia Tech, began work on a three-year bio-inspired high school engineering curriculum as part of their BIRDEE (Biologically Inspired Design for Engineering Education; Table 1) program. Integrating biology and engineering is hypothesized to increase student emotional engagement and favorably shift attitudes about science, especially biology and sustainability.

To adequately teach biom* concepts and have a transformational impact on design thinking, researchers envisioned three 8-week curricular units over three years. While the biology classroom seemed a natural starting point, the tight coupling of the existing science curriculum with standards and testing makes the integration of biom* concepts and material challenging over that duration. Thus, the engineering classroom was targeted instead, where biom* would strongly overlap with existing engineering standards and where there was more flexibility and a less well-defined curriculum with which to integrate. Teaching in the engineering classroom, however, has its own challenges for biom*.

Engineering courses in Georgia (US) fall under the purview of the CTAE (Career, Technical and Agricultural Education) program; a program usually focused on preparing students for a career after graduation. Engineering teachers come from a wide variety of backgrounds within the CTAE program. As a result, both engineering and biological content needed robust scaffolding, the latter of which would be entirely new material for the majority of CTAE teachers.

Biom*-specific training objectives included:

- familiarization with biom* as a design technique,
- integration of the processes and tools of biom* into standard design methodologies taught in high school, for instance, integrating biological inspiration into the ideation step of the standard engineering design process,
- using Structure-Function-Mechanism (SFM) analysis for understanding biology in the context of engineering,
- and conceptual transfer from biology to design.

During the summers of 2020 and 2021, four engineering teachers participated in professional training, including training on the fundamentals of biom*and training on specifics of the curriculum. Key challenges during training included integrating the biom* process into the variety of engineering design processes teachers use (3 different processes were used by teachers among the four teachers trained), teacher self-efficacy with and understanding of biological content (which included many misconceptions), and the tendency of teachers to fixate on single biological solutions in their own designs.

During curriculum development and training, BIRDEE researchers also discovered that traditional biologically inspired tools and processes required significant modification for deployment in a high school engineering classroom where students were only beginning to learn engineering, and where many engineering standards needed to be taught in conjunction with biom*. For example, when teaching the problem-driven and solution-based biologically inspired design processes in an undergraduate context, one can assume engineering students are familiar with the standard engineering design process (EDP); thus, teachers can focus on the differences between the standard process and the biom* processes. In the K-12 context, where the standard EDP is not yet known, the differences in the process can become an impediment to novice student learning. Thus researchers are forced to reconceptualize biologically inspired design processes into a format that will reinforce learning about the standard EDP while creating minimal dissonance with that learning. Researchers found such adaptation necessary for each biom*-specific tool they integrated as the curriculum was developed and presented to teachers during professional development.

Preliminary data from student pilot studies with two teachers hints that the tools and scaffolding adapted for the course can be used accurately and effectively by novice high school students and provides benefits to design thinking, complex systems thinking, problem formulation, and ideation. Anecdotal evidence from pilot teacher feedback (*n* = 2) suggests that some of these benefits may be transferable to the biology classroom.

#### 2.1.2. Deggendorf Institute of Technology

The Deggendorf Institute of Technology has been involved in teacher training and informing K-12 students about biomimetics since 2012. These trainings have taken place in cooperation with non-profit organizations, registered associations (like the bbw Group (https://www.bbw.de/en/what-we-do/ (accessed on 11 April 2022); Initiative of young scientists https://www.initiative-junge-forscher.de/; accessed on 28 April 2022) or during publicly funded projects, like the project Be Bio-inspired-new job opportunities with biomimetics (funded by the Bavarian state ministry of science and education in the years 2013–2014). The activities addressing students mostly aim at informing students about the topic, inspiring them to learn more about the topic and/or STEM topics, and encouraging them to go to university and study the topic or associated studying programs. Three students that DIT knows of attended one of the activities and did a mandatory internship at DIT. One of them studied biology afterward and, after graduation, worked on a biomimetics project at DIT. Another student who attended the biomimetics summer school studying biology now works in a biomimetics research group at a university. These individual examples show how biomimetics can have an impact when taught early and at different stages in the pipeline.

Teachers participating in training show a high interest in those offered by associations, especially when biomimetics is part of the curriculum, e.g., in 5th and 6th grade in the state of Bavaria, Southeast Germany, and they often do not know what to teach. The most important objective is to enable them to educate students in biomimetics and to be able to do biomimetics experiments with them. After taking the training (in 2012), teachers considered the training to be very motivating. They wished for more “exceptional examples” that they could showcase in class, more knowledge about research activities, specific experiments that could be taught in higher grades, and topics students could work on in seminars over a longer period. They were also interested in the link of traditional education to, e.g., biology or chemistry with biomimetics, i.e., how the topic is or could be integrated with traditional disciplinary education.

#### 2.1.3. Be Bio-inspired, Deggendorf Institute of Technology

The Be Bio-inspired project was aimed at fostering career opportunities in the STEM fields through the topic of biomimetics for primarily female students. During the project, 23 workshops, lectures, talks, and teacher training courses were held at 15 different schools. A total of 286 students were involved in the workshops, biomimetics was presented to more than 500 students at the lectures and talks, and 95 teachers from all types of schools took part in the teacher training courses. Next to the introduction of biomimetics, the focus of all events was also to present the research projects of the biomimetics working group at the Deggendorf Institute of Technology as well as study and career opportunities. A total of 46 female and 35 male students from grades 10 and 11 participated in a program evaluation survey. When asked whether they could imagine studying biomimetics, 21 female students (46%) answered yes, while 25 (54%) answered no. 15 male students answered yes (43%), 16 no (46%), and four were uncertain (11%). The students’ self-reported needs after the program were mainly: longer workshop time, more individual experimentation, more models to experiment with, more detailed information about biomimetics research, and more information on studying opportunities.

During the project, nine teachers shared their feedback in a survey. A total of six of them had been teaching the topic for one to two years, grades 5th–7th or 10th–12th, and two hadn’t taught it yet. One teacher replied that the question would not be about how long they had taught the topic, but more to emphasize that natural sciences explained the fundamental rules of technical applications. The time they had for teaching biomimetics varied from two to three hours per year, to two hours per week, up to two hours per week plus time for selectable courses. Their own expertise in the field came from their own studies (*n* = 1), external training (*n* = 9), literature (*n* = 5) and the cooperation with researchers (*n* = 3). Their main self-reported needs are more training (*n* = 4), biomimetics integrated into the curricula so that there is time and space for this topic (*n* = 2), as well as more hands-on material and models for their classes (*n* = 5).

### 2.2. Biom* Training in Higher Education

The transdisciplinary nature of biom* education presents a significant challenge to supporting post-secondary student learning because institutions are not designed to support non-traditional learning models [43,44,59]. Preliminary research by Jacobs and Wanieck (2022) and a study by von Gleich et al. (2010) suggests that learning about biological systems by non-biology students is limited or lacking until graduate school [7,60]. Most of the post-secondary and graduate biom* education opportunities are housed within existing disciplinary departments (usually engineering), which often over-emphasizes the contributions of that discipline to biom*, reducing it to an interdisciplinary practice rather than transdisciplinary. The consequences of this arrangement are still largely unknown. However, preliminary research conducted by Jacobs with designers in 2014 suggests that there are some mismatches in biological and engineering scale that could be creating barriers to successful biom* outcomes.

An international review of 19 post-secondary institutions offering biom* education by Wanieck et al. (2020) found a great variety of formats and target student groups [2]. From one-day long modules offered as curricular enhancements to semester-long biom* design courses, usually offered within engineering programs, to entire Master’s and Ph.D. programs, there is growing interest in formalizing biom* higher education. An analysis of the learning outcomes associated with these programs shows that teaching and learning biom* touches on many transferable skills in addition to biom*-specific skills and knowledge sets, making these informative for designing any number of transdisciplinary programs.

Largely missing from the review of the post-secondary curriculum was a clear delineation of what is biom*-specific factual knowledge. This supports the notion that much of the factual knowledge required is either context-specific or the students already have prior education in the required knowledge domain. Developing and Fostering such a biom*-specific knowledge base may provide a valuable service to the field. Disciplines are often characterized by their histories, discoveries, and leaders. Since much of this factual content can be found within the education outreach programs designed to inspire curious biom* enthusiasts, a concerted effort to collect and curate this knowledge will help formalize the superdiscipline.

### 2.3. Biom* Training in Industry

Industry biom* programs are grounded in general design theory, design theories by analogy and interdisciplinary design, and several post-hoc case studies from industry. These serve as guides to understanding what students will require to be successful biom* designers in industry. We can also draw upon and model education programming after academic research programs that resulted in successful biom* projects and from practitioners in the field such as Biomimicry thought-leader Janine Benyus. However, we lack clear guidance on what student-designers will face outside of “academic ivory towers” when applying biom* in real-world, highly constrained, and often profit-motivated design contexts. Since 2016, Helms has run a design consultancy focused exclusively on educating and executing biom* designs in industrial settings. Clients include well-recognized brand leaders in aerospace, personal products, retail and apparel, materials, and chemistry industries (NDA prevents disclosure of client names) and organizations such as the Innovation Research Interchange (iriweb.org, accessed on 28 April 2022).

The sponsor of biom* education or consulting services in large industrial organizations is typically associated with one of two groups within an organization: the product R & D group or the group focused on corporate sustainability. This is not to say the priorities are product innovation or sustainability. In fact, they are usually both, but internal sponsors typically emphasize one aspect more than the other, depending on their perspective. The trainers and design consultants that provide these services are either associated with organizations focused exclusively on biom* design, of which there are few, the most predominant of which is the Biomimicry Institute in Missoula, Montana, USA; with small, often individual or boutique design consulting practices, that offer biom* as a value-added service; or they are research practitioners associated with a university or university group. Clients engage biom* partners to understand the principles and practices of biom* design in one of three ways: through educational experiences such as workshops, through consultant-led product innovation projects, or through cooperative, learning-by-doing projects.

Purely education engagements may be tailored to specific product lines or domains but rarely include specific product outcomes, while product consulting and learn-by-doing engagements seek to develop specific IP for a product or domain or to identify lines of research where IP is likely to be found. In our experience, product work is typically exploratory, intended to expand the design space without necessarily arriving at a finished design during the consulting or training engagement. A finished design is unlikely. Product focused-projects often focus on well-trod and optimized product spaces, where new thinking is needed to extend the Pareto boundary (thereby increasing the number of objectives that can be maximally achieved without compromising any one objective). Once a new biological concept is identified as potentially viable, it may take several years of focused research to fully understand and integrate the new concept into existing products, materials, and manufacturing processes.

Product-focused engagements include product and scientific experts from the sponsoring organization, usually with advanced degrees and experience in their fields ranging from three to thirty years. R & D management and personnel from product-related organizations, such as brand managers and customer experience researchers, are typically included, though this can vary substantially. Their goals may range from increasing creativity through half-day workshops to months-long research activities meant to identify groundbreaking IP that changes the competitive landscape. Our experience suggests that the latter is always the ultimate and often the expected goal, even if tacit. Where groundbreaking IP is the explicit goal, because the primary audience in these activities usually consists of technically-minded product domain experts, they are best served by practitioners that can meet the client at their level of technical expertise-providing technical knowledge about biological mechanisms that their product engineers can directly translate to their design challenge. That is to say it is not enough to lead the sponsor to the biology, but one must be able to relate the biological mechanism to the practitioner from a technical perspective so that the sponsor can readily see its application to their challenge and can judge the feasibility of such an approach in their own manufacturing and capability context. Follow-up often involves the pairing of sponsors with research labs capable of conducting specialized research on or with the biological sources of inspiration. Conversely, where creativity is the goal without a specific IP goal in the engagement charter, it may be possible to conduct the engagement with a lighter technical touch and a greater focus on process than on product. Without additional follow-up, such engagements tend to inspire in the short term without producing a lasting effect. All too frequently, we hear, “we did a biomimetics workshop, but now it’s just a binder full of ideas that sits on the shelf”. The ability to pivot from inspiration to IP development is a critical follow-up skill required for successful biom* industry engagements.

In the case of sustainability-led engagements, the audience is biased toward those associated with the sustainability in the organization, and less toward deep product experts, though they are also present. We do little work in this area directly, and so refrain from speculation about the character of such engagements.

**Figure 2:**
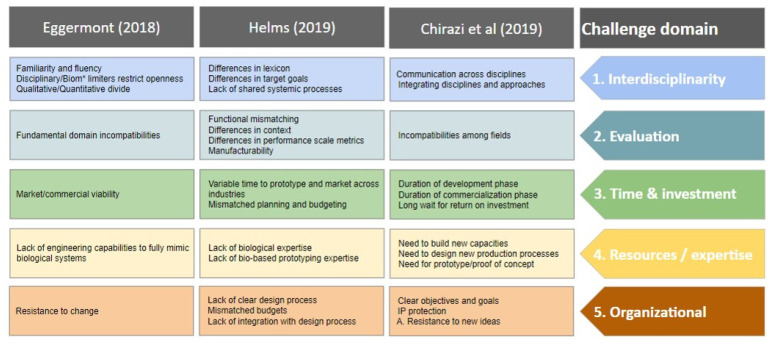
Integrative challenge domain model of industry/biom* challenges [48,61,62].

A list of common challenges experienced in these consulting engagements and gleaned from discussions and surveys conducted over the last six years was presented in Helms, 2019 [61]. Similarly, Wanieck and Jacobs surveyed industry challenges as documented in Chirazi et al. (2019), and Eggermont (2018) conducted interviews with designers to document their challenges [48,61,62]. An integration of these data shows significant overlap, and we present an integrated synthesis below (Figure 2).

## 3. Industry Challenges and Pipeline Solutions

Engaging in biom* activity involves complex research, development, and commercialization with many opportunities for failure along the way that may not be directly associated with the complexities of biomimetics per se. Though academic researchers have been focused on the challenge of knowledge transfer (i.e., [4]), it is important to account for the challenges experienced by practitioners. Here we present an integration of three sets of challenges (Figure 2), with some additional elaboration for each category. We then derive the educational implication for these challenges and identify locations along the education pipeline that may be best suited to address them.

**Figure 3:**
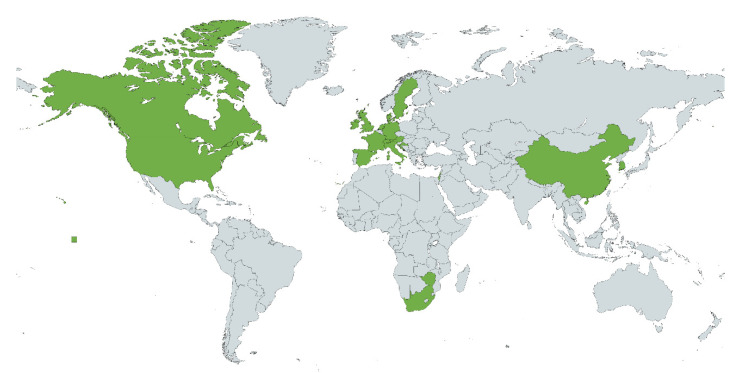
Geographical distribution of practitioners who shared industry challenges (green), based on [62]. Image created with mapchart.net, CC-BY-SA.

The Helms (2019) industry challenges were derived from five years of experience supporting industry partners in biom* design. During this time, Helms and colleagues spent hundreds of hours working on the identification, understanding, conceptual design, and quantification of new biomimetic technologies for Fortune 500 industrial partners, in addition to conducting broader industry Biom* workshops. The team documented the key challenges across multiple projects. Helms and colleagues are regular presenters with the Innovation Research Interchange (iriweb.org, accessed on 28 April 2022), an organization typically attended by R & D executives and staff from large research organizations (e.g., Fortune 500 companies, NASA, etc.), which have been providing a breadth of additional industry feedback. The Chirazi et al. (2019) challenges were derived from 14 business case studies and data collected for the BioM Innovation Database [4], including 75 interviews with designers of biom* technologies. The 75 interviews were conducted with designers from North America (*n* = 36), Europe (*n* = 30), Oceania (*n* = 6), Asia (*n* = 2), Africa (*n* = 1), as shown in Figure 3. The Eggermont industry challenges were derived from 65 interviews with designers, educators, and researchers. The 65 interviews were collected for Zygote Quarterly and using open-coding via the qualitative analysis software Atlas.ti, the following interview questions were analyzed: (1) What are your impressions of the current state of biomimicry/bio-inspired design? (2) What do you see as the biggest challenges? (3) What is your best definition of what we do? (4) What area should we focus on to advance the field of biomimicry? Here we focus our integrative analysis on the responses to question 2.

### 3.1. Industry Challenges

Each of the industry challenges were grouped within one of five challenge domains identified using an iterative process of grouping like-challenges and, through discussion and consensus, reducing the number of categories. We identified five distinct challenge domains: (1) Interdisciplinarity, (2) Evaluation, (3) Time and Investment, (4) Resources and expertise, and (5) Organizational. These categories and the examples discussed in the following section draw from our data, the majority of which was collected from industrial R & D organizations. As such, while some examples are biased toward that data, we found these challenges also occur with participants outside of industrial R & D organizations. However, they differ in degree, relative importance, and manner of expression, depending on the nature of those organizations.

*Interdisciplinarity.* The challenges associated with working on an interdisciplinary or transdisciplinary design project and/or on an interdisciplinary design team are well established and persistent. The research methods to achieve interdisciplinary biom* project goals sometimes do not exist. For example, the engineering characterization of biological materials at nanoscale levels under natural conditions is extremely challenging. In other cases, the research may require integrating methods and tools from multiple disciplines. For example, connecting signal transduction in the sensory organs of dragonflies to perception and motor control may involve biologists working with electrical and mechanical engineers, each of whom brings a different set of tools and methods to the research program. In the best case, achieving mutual understanding followed by integration of these methodologies merely slows the pace of research; worst case, they interfere with or contradict each other, and new methods may be required. Furthermore, the value placed on the quantitative and mathematical characterization of systems, typical within engineering, can create disciplinary value judgements, especially when non-quantitative methods from other domains are viewed as less rigorous or inferior. The team itself may face challenges in communicating across methodological lexicons and sometimes also have different research aims, further complicating collaboration.

*Evaluation.* Identifying suitable biological models has been a dominant research focus within the biomimetics literature. At present, most biological models are encountered through personal, and often serendipitous, circumstances. Within the BioM Innovation Database, of the 75 interviews conducted with designers, only seven reported having considered more than one biological model in their process [4]. The overwhelming abundance of biological innovations we know (or will know about) may be sufficient to support a more serendipitous approach. And yet, the challenge remains, especially as the need (e.g., climate crisis) increases. A complementary problem exists when a systematic search reveals potentially scores of biological systems that may contribute toward an improved design outcome. With limited time and resources, all potential sources of inspiration cannot be pursued. There are currently very few tools for systematically evaluating the most promising of these biological models for deeper evaluation in the context of industry constraints [63]. As the abundance of biological data grows, this problem will compound over time.

Evaluating the outcomes of a biom* design process remains elusive as well. Throughout the history of biom* design, the evaluation challenges include determining a minimum threshold above which something is deemed biomimetic, selecting criteria against which ‘success’ can be measured, and ensuring the transferability of those metrics across sectors. For example, measures of sustainability are not critical to some advocates and practitioners of biom* design, whereas, for others, it is a fundamental tenet of the approach and ought not be considered biom* design without it. Reaching the implementation phase can be correlated to using prototyping, also for making decisions in the context of considering trade-offs between, for example, practicality and sustainability or innovation and cost-effectiveness [64]. Much has been done to address these challenges, including the formation of an International Standards Organization committee (ISO/TC 266 Biomimetics; https://www.iso.org/committee/652577.html, accessed on 28 April 2022) to create an international standard for the evaluation of biomimetic technologies.

*Time and Investment.* According to one study, the average development time from concept to market in biom* is six years [48], though development time and expected timelines are domain and technology-dependent. For example, a retail shoe manufacturer that we’ve worked with expects a design-to-market turnaround of 6 months, while personal products lines that look at materials development for applications such as diapers and tissues may look at a 3–5 year time horizon, and aerospace innovators often look ten or more years along. The period between prototype and commercialization is a well-established ‘danger zone’ full of obstacles that are not necessarily biom*-specific. Not overcoming this challenge is likely a failure to recognize its importance.

*Resources and expertise.* A lack of expertise is often associated with an imbalanced design team where either the biology or the engineering (for example) are not well represented, sometimes missing entirely. Most often, it is the biological expertise that is absent (e.g., [4,11,14]), though it remains unclear the degree to which it should be represented. With larger design teams, more methods or processes, and the need to integrate research and development across several disciplines, resourcing becomes far greater than a project existing within a single domain. Team members may not fully appreciate the resourcing required outside of their own discipline. Different roles of the design team can be defined, and team members can choose which tasks to perform on their own and when to invite external expertise [10,65].

*Organizational.* The interdisciplinarity of biom* design and development amplifies the already existing organizational challenges associated with research and development. Reaching consensus on project objectives, meeting budgetary expectations, and welcoming new ideas and tools, become even more difficult to reconcile when design teams are larger and of greater disciplinary diversity.

**Figure 4:**
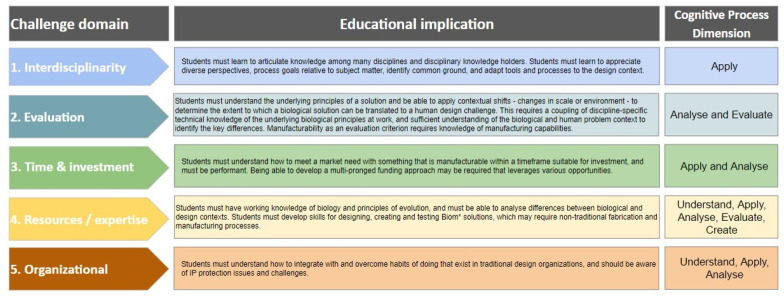
Educational implications that address the industry challenge domains.

What becomes clear with the integration of these industry challenges is that the superdiscipline of biomimetics [7] itself has advanced considerably over the past decades. Challenges experienced in the infancy of biom* focused on ‘how to do biomimetics’, and now we are challenged with how to do it efficiently, more deliberately, or more frequently. From here, we can look at how the biom* education pipeline either contributes to creating these challenges or may become a source for mitigating them by first deriving the educational implications of these Challenge Domains, shown in Figure 4.

### 3.2. Pipeline Solutions

*Interdisciplinarity.* Designers speak a language that is often unique to their discipline, filled with specialized terms with subtle meanings that often differ from common usage; the same is true of biologists. Miscommunication and misunderstanding are common and often undetected when common usage differs from technical meaning. The processes, methods, and goals of designers are different from scientists—one seeks to solve problems while one seeks to explain existing phenomena—though overlap exists. Though the challenges associated with supporting interdisciplinary research and development are found throughout institutions of higher education, there are likely pockets of interdisciplinary excellence within the biom* education pipeline. The industry challenges experienced while working across disciplines may be supported in the education pipeline where transferable skills (those discipline-agnostic skills that are not associated with any one discipline and are common to all) are taught and learned. These skills include Adaptability and Flexibility [66,67,68,69,70], Creativity [66,68,69,70,71,72], and Teamwork [66,67,68,69,71,72]. Within the pipeline, we find examples of transferable skills teaching in post-secondary education, where extended exposure to biom* topics include the opportunity to work on teams and engage in team-building experiences. From Wanieck et al. (2020), we searched the pipeline for examples where learning objectives are associated with the teaching of transferable skills (learning objectives 12, 16, and 17 from [2]). More are given too in [44]. Dr. Petra Gruber’s undergraduate course in BioDesign and Biomimicry Design Challenge courses at the University of Akron [73,74] taught students how to integrate knowledge across disciplines and work together in small teams. Both the curriculum and the learning context support the learning outcomes; open to any student from any program, this course environment is conducive to learning about interdisciplinary design and teamwork.

*Evaluation.* It is often the case that many potential biological sources can be applied to a design challenge, but which is best, and do we consider more than just one model (e.g., [4])? Where, in the design process, is a biological model or intervention required to be deemed biomimetic? How might we evaluate the outcomes of biom* research and development? To address these challenges with the educational pipeline, we look for evidence of students understanding the underlying principles of a solution, applying contextual shifts—changes in scale or environment—to understand the extent to which a biological solution can be translated to a human design challenge. This requires coupling discipline-specific, technical knowledge of the underlying principles at work and sufficient understanding of the biological and human problem context to identify the key differences. Manufacturability as an evaluation criterion requires knowledge of manufacturing capabilities. Within the existing pipeline, there are many examples where teaching and learning about biological models and evaluating the outcomes occurs, though perhaps, not always to the comprehensive level desired by students. The Evaluation Challenge Domain addresses the very core of biom* research and development; it is the ‘how’ that we have been struggling with for decades. And yet, many advancements have been made within the pipeline. In K-12 programs, students are introduced to the idea of learning from nature with exciting case studies and unforgettable hands-on activities. In post-secondary education, in courses such as Dr. Casadevall Solvas Xevi’s on Biomachines and Biomimetics [75], students are taught “how to analyze nature’s solutions for analogous problems”. Within the ‘teaching towards industry’ region of the education pipeline, Biomimicry 3.8′s Professional Training program introduces participants to many design tools and industry experts.

*Time and investment.* Industry perceives a mismatch between product time-to-market and the design and research cycles required for complete biom* development. This will vary by industry, e.g., retail time-to-market is different than aerospace, and placement in the R & D innovation ecosystem, e.g., startup time frames are different from large industrial or government labs. The educational implication of this Challenge Domain would require that students match a biom* technology with a market need that is manufacturable within a timeframe suitable for investment and must be performant. A multi-pronged funding approach may be required—for example, government funding for initial research, followed by government bridge funding, followed by venture capital funding or licensing arrangements. Within our biom* education pipeline, meeting these challenges is largely overlooked. However, Dr. Gopal Nadkarni’s Technology Based Startups course [76], as part of the Biomimicry Certificate at the University of Akron, is a rare exception where biomimetics-specific business teaching and learning are incorporated into a biomimetics education.

*Resources and Expertise.* Within-industry expertise is often long-standing, deep, and narrowly focused. Biom* requires new knowledge, new design methods, and frequently new manufacturing capabilities. Addressing this challenge within the educational pipeline is accomplished by establishing post-secondary programs in biomimetics. Entire programs for credentialing in biom* remain few but include the Certificate in Biomimicry at the University of Akron and the Master of Science in Biomimicry at Arizona State University. These programs recognize the ‘distinct’ knowledge base and the breadth of transferable skills required for practicing biom* research and development.

*Organizational.* Initial excitement over the possibility of biom* often yields new ideas, but lacking expertise leaves organizations without actionable next steps. Deeply held beliefs among experts and ossified methods of organizing and operating pose barriers to new ways of designing. The need for change is often met with resistance. The considerable effort and investment required to engage in new research and development methods is considered too great, and it may be perceived as a threat to individuals within the organization. Research-based innovation, especially when the research must be outsourced, can be expensive, time-consuming, and may create IP protection barriers when external research is required. These challenges can be met by learning to integrate with and overcome organizational habits that do not support evolving design goals. Particular attention needs to be paid to issues of IP, from design methods to the final product. Within the educational pipeline, we observe little attention or training targeted at overcoming these traditional and endemic organizational challenges.

It is worth mentioning that in industry the issue of search—that is, the evaluation of biological sources relevant to the design problem—does not rise to the top of the list of concerns (as presented in Figure 2) even though the authors experience this as a crucial part of the process that many students struggle with and is often discussed in the scientific community. We find that industry R & D experts often hold advanced degrees and, without difficulty, can find information in literature, including examples of biology that may inspire. Rather, they perceive their limitation first as one of time, and second in evaluating those biological sources, relative to their design problem and each other. In both cases, it is a matter of resource allocation and gaining the highest likely return at the lowest marginal cost. This is not to say that we ought not teach our students how to find relevant biological sources, but rather that we should increase our focus on efficiently finding sources and quickly evaluating those sources for relevance, given the constraints of the design challenge. Fayemi et al. 2017, identify a dearth of support for the process of evaluation in biom*, suggesting that, coupled with industry demand, efficient analogy evaluation may provide fruitful grounds for additional research. Likewise, considering the breadth of available process and technology support for search, and the lack of industry demand, training students on search may become progressively less valuable than providing other types of biom* design training.

## 4. Conclusions

The scientific and industrial revolutions set humanity on a path where we not only separated ourselves from nature but also actively set out to manipulate, predict, and concur with nature. This “created the basis for a reductionistic science of detached objectivism” [77]. Biom* education demonstrates to our students how to be part of nature again and what we can learn from nature and remove the historical urge to control nature, especially if it is an extended experience. The tools and methods taught introduce patterns, processes, algorithms, and applications that are already proving to be a shift in our current paradigm and will shape future biom* developments. An example is SwarmLogic^®^ by the company Encycle: an energy efficiency technology that integrates with a building’s controls to reduce electric costs. Swarm Logic controllers establish a wireless network among power-consuming appliances, enabling them to communicate among themselves autonomously. Using a custom algorithm inspired by honeybee communication, the connected appliances spread energy demand among them. In one case study, this resulted in a yearly reduction of greenhouse gas emissions by 3900 tons. A deep dive into current innovations is beyond the scope of this article, but we point the reader to [78]. As discussed earlier, biomimetics can contribute to educating a new generation of designers who have a new mindset on respecting biological models. Even if it might simply be because they hold answers to their problems concerning accessing nature as a new realm of solutions and a knowledge of how this is done in practice. This type of education needs specific content that has been discussed earlier [2], and with this manuscript, the authors encourage to include more topics based on industry needs. This could also include a focus on sustainability which almost every company faces the urge to address at present and in the future. This would give biom* educated students a uniqueness for job opportunities. However, the clear content of what is needed for education in the context of biomimetics for sustainable innovation still needs to be defined and expanded. The potential is well known [1,79,80,81]. However, it needs to be addressed specifically by using various methods or materials (e.g., [82]).

As much as the in situ cognitive observations of classrooms informed a generation of future biom* design theory research, the authors hope the challenges described in this paper may serve as guideposts for developing educational programs in biom* that seek to prepare students for existing challenges in industry.

From the perspective of industry needs, the biom* education pipeline as presented here helps educate students to particularly understand the process of biom* with the underlying principles of biological models (evaluation) so that application in real problems becomes more deliberate. Additionally, the focus of biom* higher education and training should ensure that the needs and expectations of industry are met so that integration of the topic on a daily basis in industry becomes more realistic. A more direct connection between higher education and industry is needed to overcome the discussed challenges. This asks for the engagement of non-academic partners, not only in biom* research but also in curricular design. Once the learning objectives of biom* education align with industry needs and the content taught builds a solid foundation in new biom* knowledge, well-educated students will be the “output” of the education pipeline. These students will also be able to work in inter- and transdisciplinary biom* projects, which is crucial to addressing industry challenges related to the climate crisis.

## Figures and Tables

**Table 1:** Examples of K-12 biom* education programs for students or teachers.

Program	Level	Type	Reference
Cardiovascular and Tissue Mechanics Laboratory experience	Grade 7–9	Module	[51]
Mechanics of Materials Outreach Activity	Grade 8	Module	[52]
Project STEP	Grade 10	Module	[53]
NKU Engineering Camps	High School	Summer Camps	[54]
Making Inspired by Nature	Pre-service teachers	Curriculum integrated lessons	[55]
E3 for Teachers Summer Research Program	Teachers	Summer Internship	[56]
Research Experience for Teachers Program	Teachers	Summer Internship	[57]
BIRDEE	High School Students & Teachers	Summer training	[58]

## Data Availability

Not applicable.
